# Impact of Coexistence Phenotype Between *Staphylococcus aureus* and *Pseudomonas aeruginosa* Isolates on Clinical Outcomes Among Cystic Fibrosis Patients

**DOI:** 10.3389/fcimb.2020.00266

**Published:** 2020-06-03

**Authors:** Paul Briaud, Sylvère Bastien, Laura Camus, Marie Boyadjian, Philippe Reix, Catherine Mainguy, François Vandenesch, Anne Doléans-Jordheim, Karen Moreau

**Affiliations:** ^1^CIRI, Centre International de Recherche en Infectiologie, Univ Lyon, Inserm, U1111, Université Claude Bernard Lyon 1, CNRS, UMR5308, ENS de Lyon, Lyon, France; ^2^Pediatric Pulmonology Department, Hôpital Femme-Mère-Enfant, Hospices civils de Lyon, UMR5558, Lyon, France; ^3^Institut des agents infectieux, Hospices Civils de Lyon, Lyon, France; ^4^Equipe Bactéries Pathogènes Opportunistes et Environnement, Univ Lyon, Université Claude Bernard Lyon 1, CNRS, INRAE, VetAgro Sup, UMR Ecologie Microbienne, Lyon, France

**Keywords:** cystic fibrosis, infection, *Staphylococcus aureus*, *Pseudomonas aeruginosa*, clinical outcome

## Abstract

*Staphylococcus aureus* (SA) is the major colonizer of the lungs of cystic fibrosis (CF) patients during childhood and adolescence. As patients age, the prevalence of SA decreases and *Pseudomonas aeruginosa* (PA) becomes the major pathogen infecting adult lungs. Nonetheless, SA remains significant and patients harboring both SA and PA are frequently found in the worldwide cohort. The overall impact of co-infection remains controversial. Furthermore, co-infecting isolates may compete or coexist. The aim of this study was to analyse if co-infection and the coexistence of SA and PA could lead to worse clinical outcomes. The clinical and bacteriological data of 212 Lyon CF patients were collected retrospectively, and patients were ranked into three groups, SA only (*n* = 112), PA only (*n* = 48) or SA plus PA (*n* = 52). In addition, SA and PA isolates from co-infected patients were tested *in vitro* to define their interaction profile. Sixty five percent (*n* = 34) of SA/PA pairs coexist. Using univariate and multivariate analysis, we confirm that SA patients have a less severe clinical condition than others, and PA induces a poor outcome independently of the presence of SA. Regarding co-infection, no significant difference in clinical outcomes was observed between patients with coexisting pairs and patients with competitive pairs. However, when compared to SA mono-infected patients, patients with coexisting pair presented higher frequency and length of hospitalizations and more exacerbations. We suggest that coexistence between SA and PA may be an important step in the natural history of lung bacterial colonization within CF patients.

## Introduction

Cystic fibrosis (CF) is the most common genetic disease among the Caucasian population that affects multiple organs and causes various complications associated with patient death, such as cystic fibrosis liver disease (Debray et al., 2017) (CFLD) and cystic fibrosis related diabetes (Brennan and Beynon, 2015) (CFRD). However, the first cause of morbidity and mortality in CF remains the progressive decrease in pulmonary function, leading to an obstructive syndrome (Davis, 2006). This decline is generally due to the continuous inflammation provoked by polymicrobial infections (Davis, 2006). The main clinically significant bacteria are *Staphylococcus aureus* (SA) and *Pseudomonas aeruginosa* (PA). SA infection occurs early in children and affects up to 80% of patients aged 5–19 years (Zolin et al., 2019). SA infection is responsible for increasing inflammatory markers in early childhood (Sagel et al., 2009; Gangell et al., 2011), and adolescence patients with high SA density in throat swabs present deteriorated lung function (Junge et al., 2016). However, despite the implication of SA in a worse clinical status, PA, which becomes the dominant pathogen of the respiratory tract in adulthood (Zolin et al., 2019) (up to 60% of patients > 18 years), traditionally remains the most feared pathogen due to its strong association with most severe clinical outcomes, such as more aggravated inflammation, an increase in the number of exacerbations and a decrease in forced expiratory volume in one second (FEV1) (Kerem et al., 1990; Nixon et al., 2001; Hubert et al., 2013; Ahlgren et al., 2015).

Although SA colonization decreases as patients age, SA infection concerns more than 30% of CF adults (Zolin et al., 2019). Among these cases, co-infection by PA and SA remains significant (Hubert et al., 2013). However, it is difficult to determine whether this co-infection is a transitional stage between SA alone and PA alone or whether permanent co-infections with these two bacteria exist. Several *in vitro* studies deciphered the relationships between SA and PA in a context of pulmonary co-infection (Baldan et al., 2014; Michelsen et al., 2014; Limoli et al., 2016; Hotterbeekx et al., 2017). According to recent studies, an evolution occurs regarding interaction between SA and PA clinical isolates. PA early-colonizing isolates show strong antagonism toward SA strains (Baldan et al., 2014; Michelsen et al., 2014), especially by producing anti-staphylococcal compounds, leading to a competition state (Hotterbeekx et al., 2017). However, PA isolated from chronic lung infection lacks this competitiveness and SA succeeds in coexisting durably with PA (Limoli et al., 2016; Michelsen et al., 2016; Tognon et al., 2017). We previously demonstrated that the two bacteria may cooperate to persist more easily in lungs in this context of coexistence without antagonism (Briaud et al., 2019).

Few studies have investigated the impact of SA-PA co-infection and clinical outcomes. In addition, none of them considered the interaction state (competition vs. coexistence) between SA-PA. So far, available data have shown conflicting results on the link between SA-PA co-colonization and clinical outcomes. Ahlgren et al. did not find a significant clinical difference in adult patients co-colonized with SA and PA compared to patients colonized solely by PA (Ahlgren et al., 2015). Two studies highlighted a higher respiratory decline and rate of hospital admission for patients infected by PA alone in comparison with patients co-infected by SA-PA or by SA alone (Hubert et al., 2013; Cios et al., 2019). Finally, other studies reported that SA-PA co-infection is associated with a worse clinical outcome (Hudson et al., 1993; Sagel et al., 2009; Gangell et al., 2011; Limoli et al., 2016).

The aim of this study was to better characterize (i) co-infected patients with demographic data (age, BMI, gender), and (ii) the interaction state (competition or coexistence) between the two bacterial species. We also examined the consequence of SA-PA co-infection and SA-PA interaction state (competition vs. coexistence) on pulmonary functions (FEV1%) and clinical outcomes (e.g., number of exacerbations, number of hospitalizations). By computing demographic and clinical data with pulmonary infectious status, we gain more information on the impact of SA and PA infections and interactions on CF patients' health.

## Materials and Methods

### Patients

The clinical and bacteriological data of CF patients supervised at the two CF Centers in Lyon, France (CRCM: Center de Ressources et de Compétences de la Mucoviscidose) were collected from February 2017 to August 2018.

The inclusion criterion was a stable microbiological status for SA and /or PA colonization, defined by the following criteria: (i) at least three respiratory samples collected for each patient throughout the period considered; (ii) at least 2 months between two successive samples; (iii) all the samples collected during the study for a patient had the same status with respect to the presence of SA and / or PA. The patients who did not match the criteria, or who were not co-colonized by SA or PA were excluded.

This study was submitted to the Ethics Committee of the Hospice Civil de Lyon (HCL) and registered under CNIL No 17-216. All the patients were informed of the study and did not oppose the use of their data.

### Clinical Data Collection

Clinical data were extracted from computerized medical files (Easily®). The data collected were: gender, age at the time of the last sampling, CFTR genotype classified between severe and moderate genotype regardless of clinical severity (5, 19), pancreatic insufficiency defined by fecal elastase <200 μg/g, CF-related diabetes (CFRD) or a carbohydrate intolerance, cirrhosis, need for long term oral or enteral supplementation and/or undernourishment (defined as a body mass index (BMI) (weight/height^2^) lower than 17 kg/m^2^ in an adult and −2 standard deviations (SD) in a child according to gender and age). The pulmonary function was defined by the FEV1 expressed as a percentage of the predicted value (%pred). We also collected the number of hospitalizations, the length of hospital stays and the number of exacerbations during the 9 months preceding the last sample.

### Microbiology

The microbiological composition of each respiratory sample was determined by the Institute for Infectious Agents, HCL. The interaction state (coexistence or competition) of SA-PA pairs was defined by agar competition assay as previously described (Briaud et al., 2019). Briefly, SA and PA isolates recovered from co-infected patients' expectorations were cultured in 10 ml of BHI, at 37°C, 200 rpm. From overnight cultures, SA and PA suspensions were diluted to OD_600nm_ =0.5. Then, 100 μL of SA suspension was spread uniformly onto trypticase soy agar (TSA) plates. After drying, 5 μl of PA suspension were spotted at the center of the plates. The plates were incubated at 37°C for 24 h. The competitive phenotype was characterized by an inhibition halo of SA growth. In the absence of inhibition halo, isolates were defined in coexistence.

Based on these microbiological analyses, the patients were categorized into four groups: (i) SA alone, (ii) PA alone, (iii) SA-PA in competition, and (iv) SA-PA in coexistence.

### Statistical Analysis

Two different analyses were performed using the same process: (i) SA vs. PA vs. SA+PA, and (ii) SA vs. PA vs. SA+PA in coexistence vs SA+PA in competition.

Factor Analysis of Mixed Data (FAMD) was used for initial data screening. Then, univariate analysis was performed to determine significant differences between groups. For continuous variables (age, BMI, FEV1, number of hospitalizations, length of hospitalization, and number of exacerbations), Kruskal-Wallis tests were used to identify whether one population was different from the others. Afterwards, to individually test each pair of populations with significant Kruskal-Wallis tests, a Mann-Whitney Wilcoxon tests was used. Fisher's exact tests were performed to compare categorical variables (CFTR genotype, gender, denutrition, pancreatic insufficiency, CFRD, liver cirrhosis, enteral nutrition, and oral food supply). For multiple comparisons, tests were corrected by a Bonferroni method and statistical significance was set with a q-value threshold at 0.05. Finally, a multinomial log-linear model [nnet package (William, 2016)] was used to determine significant associations between infectious status groups and variables selected using the Akaike information criterion (AIC) (Akaike, 1979). The AIC analysis was performed to identify the smallest and fittest set of variables describing our data. An adjusted odds-ratio with a 95% confidence interval was reported for each final variable. All the analyses were performed using R v3.5.3 (R Core Team, 2018).

## Results

### Clinical Characteristics of Patients

Of the 655 CF patients monitored in Lyon hospitals, we selected patients with at least three respiratory specimens with SA and/or PA during the study period. Two seventy six patients were excluded because of the absence of the three samples required. Sixty-five patients had neither SA nor PA infection. Finally, 48 and 54 patients were excluded because of unstable SA-PA co-colonization during the period or the inability to assess their pulmonary function (age <4 years). For co-infected patients, we excluded five patients because they presented several PA isolates with both competitive and coexisting interaction phenotypes with SA. Finally, 212 patients with CF were included in this study.

The cohort consisted of 124 adults and 88 children with a mean age of 21.70 years (range 4–69). The male-female ratio was homogeneous with ~51.42% (*n* = 109) of men. About 80.66% (*n* = 171) had a severe CFTR genotype and 41 patients had a moderate one. During this period, 39.62% (*n* = 84) were subject to exacerbation and 25.94% (*n* = 55) of the patients were hospitalized (Table S1).

The 212 patients were classified following their chronic bronchial colonization into 3 different groups: SA alone, PA alone and SA+PA. Co-infected patients were split into 2 subclasses (competition and coexistence) regarding the SA-PA interaction state determined by agar competition assay (Table 1). Among the 212 patients, 52.83% (*n* = 112) had chronic bronchial colonization with SA, 22.64% (*n* = 48) with PA and 24.53% (*n* = 52) with SA+PA. Within the 52 co-infected patients, 65.38% (*n* = 34) of SA and PA isolates presented a coexistence interaction and 34.62% (*n* = 18) presented a competition phenotype. Patients colonized solely with PA were older (average of 32.02y) than co-infected patients (average of 23.38y) and the latter were older than patients colonized by SA (average of 16.49y) (Table 1). This is consistent with the natural history of infections and international values (4).

**Table 1:** Clinical characteristics of CF patients according to their bacteriological status.

	**SA alone (%)**	**PA alone (%)**	**SA and PA**			
			**Competition**	**Coexistence**	**Merge**	p_value^*^
Number	112/212 (52.83)	48/212 (22.64)	18/52 (34.61)	34/52 (65.38)	52/212 (24.53)	
Age (years)	16.49 ± 8.77	32.02 ± 13.82	23.67 ± 8.98	23.24 ± 10.52	23.38 ± 9.93	<0.0001^a^
≥18 years	45/112 (40.18)	42/48 (87.50)	14/18 (77.78)	23/34 (64.65)	37/52 (71.15)	
Sex, male	63/112 (56.25)	23/48 (47.92)	10/18 (55.6)	13/34 (38.24)	23/52 (44.23)	ns
Genotype						
Moderate	26/112 (23.21)	9/48 (18.75)	3/18 (16.67)	3/34 (8.82)	6/52 (11.54)	ns
Severe	86/112 (76.79)	39/48 (81.25)	15/18 (83.33)	31/34 (91.18)	46/52 (88.46)	
BMI (kg/m^2^)	18.06 ± 2.68	20.74 ± 2.42	19.25 ± 2.56	19.71 ± 3.56	19.55 ± 3.23	<0.0001^a^
Undernourishment	11/112 (9.82)	1/48 (2.08)	1/18 (5.56)	3/34 (8.82)	4/52 (7.69)	ns
Oral food supplementation	23/112 (20.54)	13/48 (27.08)	3/18 (16.67)	15/34 (44.12)	18/52 (34.62)	ns
Enteral nutrition	4/112 (3.57)	0/48 (0)	0/18 (0)	4/34 (11.76)	4/52 (7.69)	ns
Pancreatic insufficiency	101/112 (90.18)	45/48 (93.75)	17/18 (94.44)	33/34 (97.06)	50/52 (96.15)	ns
CF-related diabetes	9/112 (8.04)	14/48 (29.17)	3/18 (16.67)	10/34 (29.41)	13/52 (25.00)	0.0007^b^
Cirrhosis	6/112 (5.36)	2/48 (4.17)	0/18 (0)	1/34 (2.94)	1/52 (1.92)	ns
Hospitalizations						
Number	17/112 (15.18)	18/48 (37.50)	6/18 (33.33)	14/34 (41.18)	20/52 (38.46)	0.0003^a^
Length	8.00 ± 6.10	17.39 ± 14.39	7.33 ± 6.12	29.14 ± 27.39	22.60 ± 25.06	0.0002^a^
Number of exacerbations	0.25 ± 0.69	1.44 ± 1.37	1.28 ± 1.60	1.35 ± 1.50	1.33 ± 1.52	<0.0001^a^
FEV1 (% predicted)	85.87 ± 22.39	55.09 ± 18.64	59.72 ± 18.73	64.59 ± 21.79	62.90 ± 20.73	<0.0001^a^

*BMI, Body Mass Index; FEV1, Forced Expiratory Volume in one second*.

* *P values from comparison between three groups: SA vs. PA vs. SA+PA (merge)*.

a *P values from Kruskal-Wallis non-parametric test for comparison of the three groups (continuous variables)*.

b *P values from Fisher's exact test for comparison of the three groups (categorical variables)*.

We proceeded to an FAMD analysis to decipher the similarity and heterogeneity between patient groups using both continuous and categorical variables. The first two axes retained accounted for 39.47% of the total variance of the data (Figure S1). Four variables contributed to the first axis: number of exacerbations, number and days of hospitalization and FEV1. Age, BMI and not having pancreatic insufficiency contributed to the second axis. The distribution of the 212 patients based on the two first dimensions overlapped between the 4 groups (Figure S1). Patients infected by SA were the most represented group and appeared to have more homogeneous clinical variable values than other patients. On the contrary, patients co-infected in a coexistence state seemed to have values with greater heterogeneity (Figure S1).

### Impact of Co-infection on CF Patient Clinical Outcome

**Figure 1:**
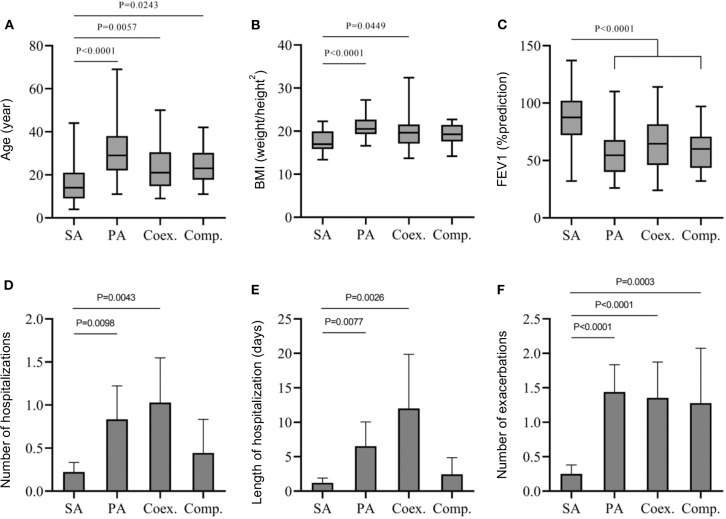
Comparison between SA mono-infected (SA), PA mono-infected (PA) and co-infected groups in competition (Comp) or in coexistence (Coex) for age mean **(A)**, BMI **(B)**, FEV1 **(C)**, number of hospitalization **(D)**, length of hospitalization **(E)**, and number of exarcerbations **(F)**. A Mann-Whitney Wilcoxon non-parametric test corrected by a Bonferroni method was used.

To define whether co-infection was associated with poorer clinical outcome, we compared three groups: patients infected with SA, patients infected with PA and patients co-infected with SA-PA (Figure 1). Continuous variable analysis differentiated the 3 patient groups (Table 2). SA patients seemed to be younger than co-infected patients, the latter being younger than PA patients (*p* < 0.0001). The SA group stood out from the others due to lower BMI values (PA vs. SA: *p* < 0.0001, SA+PA vs. SA: *p* = 0.0074), a smaller number of hospitalizations (PA vs. SA: *p* = 0.0017, SA+PA vs. SA: *p* = 0.0013), a reduced length of hospitalizations (PA vs. SA: *p* = 0.0011, SA+PA vs. SA: *p* = 0.0010), a lower number of exacerbations (*p* < 0.0001) and a higher FEV1 (*p* < 0.0001). Co-infected and PA groups were not statistically different for the number of hospitalizations or length of hospitalizations, the number of exacerbations or FEV1. Only age (*p* = 0.0012) and BMI (*p* = 0.029) values were significantly different. For the analysis of categorical variables, CFRD was only statistically significant between the PA vs. SA mono-infected groups (*p* = 0.0031) and co-infected patients vs. SA (*p* = 0.0169) (Table S2).

**Table 2:** P-values for continuous variable comparisons between SA mono-infected (SA), PA mono-infected (PA) and co-infected groups (SA+PA).

	**PA vs. SA**	**SA+ PA vs. SA**	**SA+PA vs. PA**
Age	<0.0001	<0.0001	0.0012
BMI	<0.0001	0.0074	0.029
Number of hospitalizations	0.0017	0.0013	ns
Length of hospitalization	0.0011	0.0010	ns
Number of exacerbations	<0.0001	<0.0001	ns
FEV1	<0.0001	<0.0001	ns

*A Mann-Whitney Wilcoxon non-parametric test corrected by a Bonferroni method was used*.

**Figure 2:**
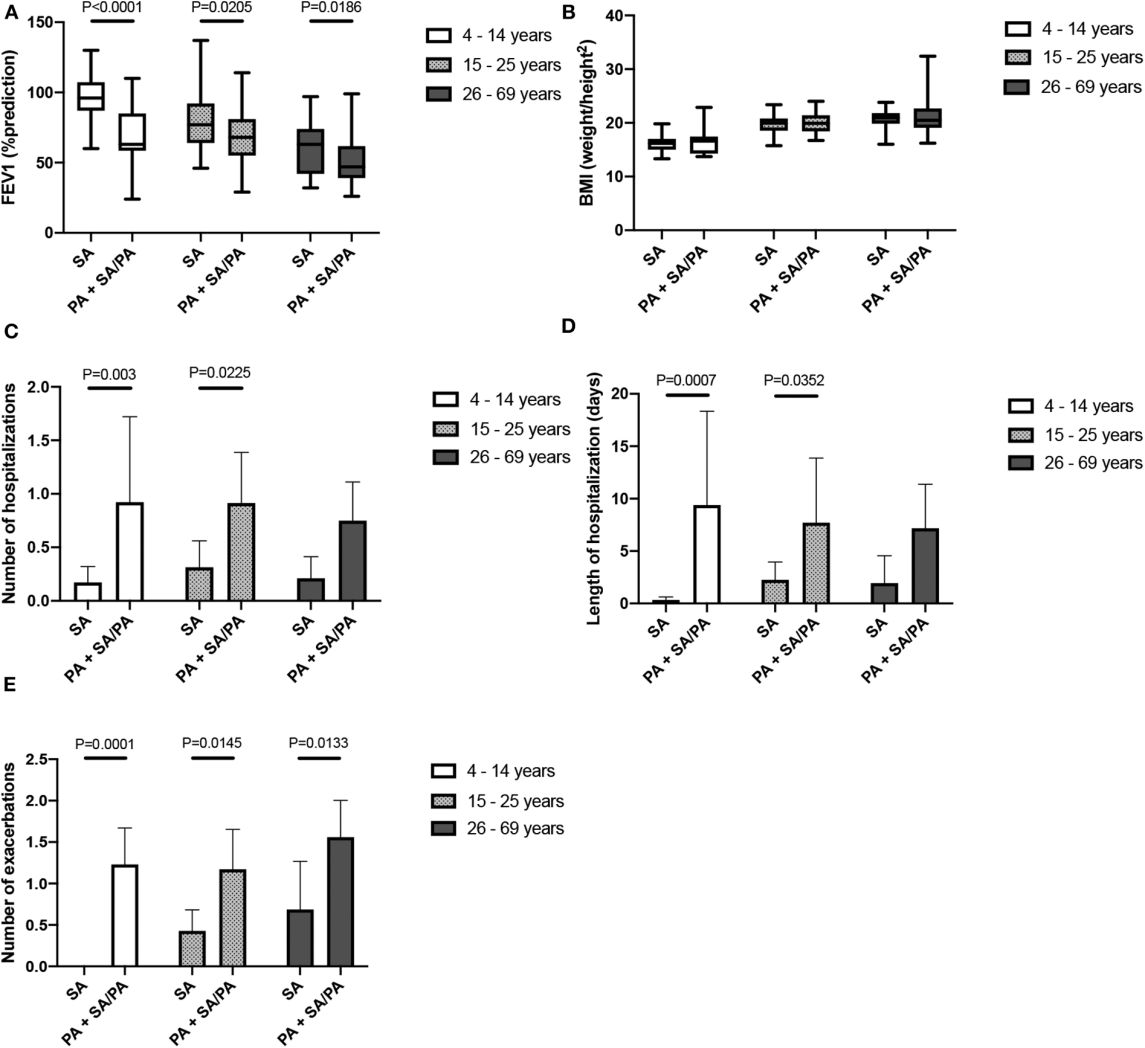
Comparison between SA mono-infected (SA) and PA mono-or co- infected (PA) groups within three age classes of patients. FEV1 **(A)**, BMI **(B)**, number of hospitalization **(C)**, length of hospitalization **(D)** and number of exarcerbations **(E)** are represented. A Mann-Whitney Wilcoxon non-parametric test was used.

Since the PA group appears to have the worst clinical outcome but also corresponds to the oldest group of patients, it is questionable whether the deterioration of the clinical condition of the patients is linked to the age or presence of PA. To answer this question, we classified the patients into three age groups (4–14, 15–25, and 26–69) and analyzed the impact of PA infection within these different groups on continuous variables (Figure 2). We showed that PA patients had significantly lower FEV1 and a higher number of exarcebations for all three age groups. We also observed a higher number and length of hospitalization for two of the age classes. Therefore, we concluded that the poor clinical outcome can be independently attributed to PA colonization and not to the age of the patients.

To further investigate these results and consider the potential association between variables, multinomial analysis with infection type as outcome was performed using age, BMI, FEV1, number of exacerbations, number of hospitalizations, length of hospitalizations and CFRD (Table 3). The aim of the multivariate analysis was to obtain only one set of variables which fit the entire dataset between groups. This was performed in order to compare odds ratio inside each comparison. Two kinds of variables were eliminated using AIC criteria: variables which could clearly not explain the outcome and variables which were correlated to each other.

**Table 3:** Adjusted odds ratios of cystic fibrosis patients' infection status.

	**PA vs. SA**		**SA+** **PA vs. SA**		**SA+PA vs. PA**	
	**OR (95 % CI)**	**p_value**	**OR (95 % CI)**	**p_value**	**OR (95 % CI)**	**p_value**
Age	–	0.1354	–	0.6767	0.9477 (0.9017, 0.9961)	0.0345
BMI	1.2662 (1.0313, 1.5545)	0.0242	1.2022 (1.0015, 1.4432)	0.0481	–	0.5913
Number of exacerbations	2.0282 (1.2559, 3.2755)	0.0038	2.1460 (1.3514, 3.4078)	0.0012	–	0.7298
FEV1	0.9612 (0.9355, 0.9877)	0.0043	0.9701 (0.9478, 0.9930)	0.0108	–	0.4947

*Multinomial regression analyses were performed to study associations between the type of infection [SA mono-infected (SA), PA mono-infected (PA) and co-infected groups (SA+PA)] and clinical outcomes. Adjusted odds ratios with 95% confidence intervals (CI) are mentioned*.

In this context, variables about hospitalizations were not kept in the final model due to their correlation with the number of exacerbations [Cor (95% CI): 0.6648 (0.5822, 0.7338) (*p* < 0.0001)], the number of hospitalizations and [Cor (95% CI): 0.5843 (0.4879, 0.6666) (*p* < 0.0001)] and the length of hospitalizations.

The final model demonstrated that patients infected with PA were more likely to have a high BMI [OR (95% CI): 1.2662 (1.0313, 1.5545)], a higher number of exacerbations; [OR (95% CI): 2.0282 (1.2559, 3.2755)], and lower FEV1 [OR (95% CI): 0.9612 (0.9355, 0.9877)] compared to patients infected with SA (Table 3). The results were similar when the co-infected group was compared to SA only [OR (95% CI)]: BMI: 1.2022 (1.0015, 1.4432); exacerbations: 2.1660 (1.3514, 3.4078); FEV1: 0.9701 (0.9478, 0.9930)]. When comparing the SA-PA group to the PA group, only age criteria was significant as co-infected patients were more likely to be younger than PA mono-infected patients [OR (95% CI): 0.9477 (0.9017, 0.9961)].

### Impact of Bacterial Coexistence and Competition Within Co-infected CF Patients

The second objective of this study was to determine whether the interaction profile between SA and PA could affect patient clinical outcome. Thus, we compared the mono-infected groups (SA only and PA only) with two co-infected groups: SA plus PA in coexistence and SA plus PA in competition.

Using continuous variables, the comparison between coexistence and competition groups showed no significant difference (Figure 1). Furthermore, no statistical differences were found between PA mono-infected or SA plus PA in coexistence or in competition. Compared to SA groups, both SA plus PA interaction groups seemed to infect older patients (Coex. Vs. SA.: *p* = 0.0057; Comp. vs. SA: *p* = 0.0243) with a higher number of exacerbations (Coex. Vs. SA: *p* < 0.0001; Comp. vs. SA: *p* = 0.0003) and lower values of FEV1 (Coex. Vs. SA: *p* < 0.0001; Comp. vs. SA: *p* = 0.0001). However, it is noteworthy that, for BMI, number of hospitalizations and length of hospitalization criteria, only the coexistence group differs significantly from the SA groups (Figure 1). Thus, the coexistence group presented higher BMI values (*p* = 0.0449), a higher number of hospitalizations (*p* = 0.0043) and longer hospitalization stays (*p* = 0.0026) compared to the SA groups.

Considering categorical variables, no characteristics were statistically significant between the two types of interaction (Table S3). However, when comparing the SA group, there was a higher number of CFRD patients in the coexistence group (*p* = 0.0164); these criteria were not significant when the SA group was compared to the competition group.

Finally, multinomial analysis was performed using all the significant characteristics from the univariate tests. The final predictors retained after analyses of deviance through AIC criteria were BMI, need for oral food supplementation, number of exacerbations and FEV1. A significant difference was found between the two types of interaction in co-infected patients. The coexistence state group was more likely to need oral food supplementation than the competition group [OR (95% CI): 0.2262 (0.0536, 0.9541)] (Table 4). As with the univariate results, the multivariate analyses confirmed that patients co-infected by either a coexisting SA-PA pair or a competition pair had higher odds of having more exacerbations than those infected only by SA [OR (95% CI): 2.2896 (1.3982, 3.7494): 1.9090 (1.0956, 3.3262), respectively]. Compared to the SA group, the competition state group seemed to have lower values of FEV1 [OR (95% CI): 0.9589 (0.9300, 0.9887)] whereas the coexistence state group was more likely to have higher BMI [OR (95% CI): 1.2047 (1.0210, 1.4213)].

**Table 4:** Adjusted odds ratios of cystic fibrosis patients' infection status.

	**Coex vs. SA**		**Comp vs. SA**		**Comp vs. Coex**	
	**OR (95 % CI)**	**p_value**	**OR (95 % CI)**	**p_value**	**OR (95 % CI)**	**p_value**
BMI	1.2047 (1.0210, 1.4213)	0.0273	–	0.3199	–	0.4872
Oral food supplementation	–	0.0801	–	0.3609	0.2262 (0.0536, 0.9541)	0.0430
Number of exacerbations	2.2896 (1.3982, 3.7494)	0.0010	1.9090 (1.0956, 3.3262)	0.0228	–	0.4472
FEV1	–	0.0861	0.9589 (0.9300, 0.9887)	0.0072	–	0.1885

*Multinomial regression analyses were performed to study associations between the type of infection [SA mono-infected (SA), PA mono-infected (PA) and co-infected groups in coexistence state (Coex.), or in competition state (Comp.)] and clinical outcomes. Adjusted odds ratios with 95% confidence intervals (CI) are mentioned*.

### Diabetes Outcome and Infectious Status Are Not Associated

Additional multivariate analyses were performed on CFRD patients due to a statistically significant difference between groups when performing univariate analyses (Tables S2, S3). First of all, a multinomial analysis was conducted with all the variables (including infection status) and taking CFRD as outcome. On the basis of the analyses of deviance conducted with AIC criteria, the following variables were excluded from the multinomial analysis: sex, BMI, undernourishment, enteral nutrition, cirrhosis, length of hospitalization, number of exacerbations, FEV1, and type of infection. Thus, none of these variables were associated with CFRD and CFRD patients were not more likely to be infected by SA or PA, or co-infected by SA plus PA. Five characteristics were conserved in the final model in which odds ratios and associated adjusted p_values were reported (Table 5). CF-related diabetes patients were more likely to be older [OR (95% CI): 1.1013 (1.0543, 1.1504)], to need more oral food supplemenation [OR (95% CI): 3.0259 (1.2297, 7.4459)] and were more hospitalized [OR (95% CI): 2.1662 (1.5254, 3.0763)].

**Table 5:** Adjusted odds ratios of cystic fibrosis-related diabetes (CFRD) patients' infection status.

	**OR (95 % CI)**	**p_value**
Age	1.1013 (1.0543, 1.1504)	<0.0001
Genotype	–	0.1022
Oral food supplementation	3.0259 (1.2297, 7.4459)	0.0160
Pancreatic insufficiency	–	0.8192
Number of hospitalizations	2.1662 (1.5254, 3.0763)	<0.0001

*Multinomial regression analyses were performed to study associations between cystic fibrosis-related diabetes (CFRD) and other clinical outcomes including infection type. Adjusted odds ratios with 95% confidence intervals (CI) are mentioned*.

## Discussion

The lungs of CF patients are colonized by multiple bacteria and several studies were conducted to decipher the impact of these colonisations on clinical outcomes. We focused our study on the two major pathogens responsible for chronic colonization: *Staphylococcus aureus* and *Pseudomonas aeruginosa*. There is no gold standard to define chronic colonization, especially for SA. For PA infection, Leeds criteria (Lee et al., 2003) are sometimes used and define “chronic” as being when more than 50% of the preceding 12 months were PA culture positive. This definition requires frequent expectoration sampling. Furthermore, these criteria may lack sensitivity and are not always considered by clinicians (Hoo et al., 2018). Alternatively, PA serology appears to be a sensitive criterion for classifying PA infection, especially in non-expectorating children, but is not always available (Taccetti et al., 2019). In order to clearly study the impact of SA and/or PA infection, we focused on stabilized infections. Therefore, we considered chronic colonization when at least 3 samples over a 9-month period had the same colonization pattern. These criteria are close to the definition of European Consensus Criteria for PA colonization. However, this stringent method led to the exclusion of 33% of our cohort; 10% of patients were also excluded due to a change in colonization status during the period studied. Finally, in our study, 52.83% of the remaining patients had chronic SA colonization, 22.64% chronic PA colonization and 24.53% had chronic co-colonization with both bacteria. These data are consistent with those of previous studies (Hubert et al., 2013; Cios et al., 2019).

The first point of our study was to compare the impact of chronic colonization by SA alone, PA alone and SA plus PA on the clinical outcome of CF patients. Indeed, being able to evaluate (or even predict) the risks that a patient incurs depending on the type of bacterium and/or on bacterial associations (in particular between PA and another pathogen) that colonize the lungs, remains one of the major challenges in the management of patients. Thus, identifying patients with harmful bacterial associations would be of clinical interest as they have increased risks of a worse clinical outcome. These bacterial associations could become a therapeutic priority and be eradicated by targeting, for example, one of the pathogens. Conversely, we could speculate that co-infection between PA and another bacterium represents a milder step in disease evolution, probably an intermediate stage between SA alone and PA alone.

Our results showed that SA chronic colonization led to a higher FEV1 (85.87 ± 22.39), fewer exacerbations (0.25 ± 0.69), a smaller number of hospitalizations (15.18% of SA patients) and shorter stays (8.00 ± 6.10) than SA plus PA co-infection or PA alone. We did not observe any differences regarding clinical status between patients co-infected by PA and SA and those infected by PA alone. This suggests that the SA plus PA combination does not lead to over or under morbidity compared to the presence of PA alone in the lungs, and that there is no synergism in term of pathogenicity. As soon as PA colonizes the CF patients' lungs, the clinical condition of the patients appears to deteriorate, whether SA is present or not. These results are in accordance with previous results from Ahlgren et al. that demonstrate by univariate analysis that there is no difference between PA mono-infected and PA plus SA co-infected patients (Ahlgren et al., 2015). However, they are in contradiction with others. Indeed, Hudson et al. (1993) and Sagel et al. (2009) conducted similar analyses on pediactric cohort patients and showed that SA plus PA co-infections led to the worst spirometry (measured by the forced expiratory flow or FEF) (Hudson et al., 1993), a 10 years survival of 57% lower (Hudson et al., 1993) and have the highest measurement of inflammation (Sagel et al., 2009) compared to the PA group. Gangell et al. (2011) also depicted that the inflammatory response to co-infection was greater but not significantly different from mono-infection. Similarly, studies conducted by Limoli et al. (2016) and Cios et al. (2019) on mixed patient cohorts (pediactric and adults) pointed out that (i) SA plus PA co-infections were responsible for more frequent exacerbations and lower FEV1 compared to the PA group (Limoli et al., 2016) and (ii) patients mono-infected by PA were more transplanted with a higher mortality rate, the highest rates of hospitalization, annual readmission and length of stay and the shortest time to subsequent hospitalization (Cios et al., 2019). These discrepancies within studies may reflect variabilities among cohorts (size, age…), and statistical analysis and models used. However, under our conditions, where we are studying stabilized mono or co-infections, it appears that there are no differences between PA mono-infected and PA plus SA co-infected patients.

Other studies analyzed the impact of co-infection based on the presence of methicillin-susceptible *Staphylococcus aureus* (MSSA) or methicillin-resistant *Staphylococcus aureus* (MRSA). Patients co-infected by MSSA plus PA presented better outcome than infected by PA only (higher FEV1, higher forced vital capacity (FVC) and lower number of intraveneous antibiotics per year than those) (Hubert et al., 2013). Their univariate analysis showed that no difference could be established between the co-infection group and the PA mono-infection group. On the contrary, co-infection by MRSA plus PA were as severe as the PA infection (Hubert et al., 2013). Furthermore, linear regression models adjusted for gender, age at baseline, age at diagnostic, genotype, and pancreatic sufficiency in this same study showed that the MRSA/PA patients had the largest yearly decline in FEV1. This last result was confirmed by Maliniak et al. (2016). In our cohort, only 15 MRSA cases were reported. This low number of cases (9%−5/164) is slightly higher than the MRSA rate in France, which is 6.3% according to the French cystic fibrosis register, 2017. However, it was not enough to perform analyses and conclude on the impact of methicillin-resistance status on clinical outcomes. Further studies are required to figure out why MRSA—*per se* or as a surrogate marker of other genetic determinants—negatively impacts the clinical outcome of CF patients.

CFRD is a severe complication of CF and its association with bacterial infection could be used as a predictive marker of severity. CFRD seemed to be overrepresented in mono-infected PA (29.17%) and co-infected (25%) patients and under-represented in mono-infected SA patients (8.04%). CFRD represented 16.98% of our total patients and was slightly higher than the CFRD rate in the European population (Kerem et al., 2014). Limoli et al. (2016) showed that CFRD status was marginally associated with co-infection by both SA and PA [OR (95% CI): 1.90 (0.99, 3,6)] in comparison to SA or PA mono-infection. We did not confirm this result. Indeed, our multivariate analysis demonstrated that CFRD was associated with the age of the patients [OR (95% CI): 1.1013 (1.0543, 1.1504)]. As co-infected and PA infected patients are older than SA patients, CFRD could be more represented in the first two groups. However, we cannot exclude that the difference between the two studies can be explained by a lower number of patients in our study.

The second objective of the present study was to evaluate the impact of the type of interaction between SA and PA (competition or coexistence) on clinical outcomes. Among the 52 patients co-colonized with SA and PA, 65.38% of strain pairs were in coexistent interaction and 34.61% in competition. These competitive and coexistence statuses have already been described (Baldan et al., 2014; Michelsen et al., 2014; Cigana et al., 2017; Limoli et al., 2017; Tognon et al., 2017; Briaud et al., 2019). However, this is the first time that this type of interaction was evaluated in a large number of clinical isolates and we described that coexistence is the predominant interaction type in chronically co-colonized patients.

It has long been recognized that PA could suppress SA growth by several mechanisms (Hotterbeekx et al., 2017). These data led to the theory that PA colonization causes SA elimination in patients' lungs. Indeed, in our cohort, 24% of patients were co-colonized, which is consistent with other studies (Hubert et al., 2013; Cios et al., 2019). Moreover, SA-PA pairs from co-infected patients were mostly coexisting. Thus, our study shows that in 34 patients, chronic co-colonization may be due, at least in part, to the inability of PA to suppress SA. It has been well-described that in CF lungs, PA strains evolve and adapt from virulent early-infecting strains to less-virulent late-infecting strains (Michelsen et al., 2016; Limoli et al., 2017). Stresses induced by the pulmonary environment (oxidative and osmotic), antibiotic treatments, the immune system and other bacteria species, constitute selective pressure forces for the selection of adaptive mutations (Baldan et al., 2014; Michelsen et al., 2014; Wakeman et al., 2016). Virulence changes could also reduce the antagonistic action of the PA isolates on SA (Baldan et al., 2014; Michelsen et al., 2014). However, of all the factors affecting the evolution of PA in the pulmonary environment, the role of SA in PA evolution remains to be explored.

Nonetheless, in the present study, we did not observe a difference in age between patients colonized with coexisting isolates (23.24 ± 10.52) and patients colonized with competitive pairs (23.67 ± 8.98). This suggests that there may be no link between adapted late-infecting strains and coexistence. In order to clarify this point, longitudinal studies will be necessary to evaluate the link between bacteria interaction status and the evolution of isolates.

How PA tolerance toward SA may contribute to worse clinical outcomes is a point that has not been explored before. The analyses showed no significant difference in clinical outcomes between patients with coexisting pairs and patients with competitive pairs, except for the need of oral food supplementation observed with multinomial analysis.

Nevertheless, we highlighted several disparities between coexistence and competition groups when compared to SA mono-infected patients. The univariate analysis indicated that both groups were more likely to have a lower FEV1 and higher number of exacerbations. The multinomial analysis confirmed that patients with a competitive pair had a higher number of exacerbations and a lower FEV1, in comparison to SA group. However, we noticed that multinomial analyse did not confirm the lower FEV1 for the coexistence group in comparison to SA. Indeed, the number of exacerbations was correlated with FEV1. The correlation for the coexistence group [Cor (95% CI): −0.6159 (−0.7896, −0.3508) (*p* = 0.0001)] was stronger than that for the competition group [Cor (95% CI): −0.5462 (−0.8072, −0.1065) (*p* = 0.0190)]. The differences in correlation's strength could explain why multinomial results showed a significant lower FEV1 value for SA plus PA in competition state compared to SA mono-infected patients, unlike the analysis between SA plus PA in coexistence state compared to SA mono-infected patients.

More importantly, univariate analysis demonstrated that only patients with a coexisting pair were more likely to have higher BMI, number and length of hospitalizations than SA mono-infected group. This increased BMI was confirmed by multinomial analysis and might be the results of the long term oral food supply, which was more frequent in coexistence group. Multinomial analysis also indicated that coexistence group presented more exacerbations than the SA group, consistent with previous results about hospitalizations.

Overall, these results suggest that patients with coexisting pair required food supply more frequently than patients with competitive pair. Moreover, when compared to SA mono-infection, co-infection with coexisting PA appears to be more severe than co-infection with competitive PA (higher number and length of hospitalizations and more exacerbations).

Several quorum-sensing dependent virulence factors are produced by competitive PA strains such as rhamnolipids and phenazines (Hotterbeekx et al., 2017), that can trigger a higher inflammatory response leading to a lower FEV1. On the contrary, coexisting PA strains lack the production of virulence factors and should be less harmfull for the lung environment (Yang et al., 2011; Michelsen et al., 2014). The clinical outcome is therefore mainly due to other genotype/phenotype traits of PA. PA adaptation, is related to many environmental factors, including the surrounding bacteria (Yang et al., 2011; Winstanley et al., 2016; O'Brien and Fothergill, 2017). Thus, in the context of coexistence, current analysis of the impact of SA on the PA transcriptome and growth phenotype suggests that SA could favor PA persistence (Camus et al., in revision). Moreover, we previously demonstrated that PA can enhance SA antibiotic resistance (Briaud et al., 2019). Therefore, we suggest that cooperation between the two species in the coexistence state may be slightly more deleterious for the host than in the competition state. A larger patient cohort would be necessary to evaluate the real impact of the coexistence state on clinical outcomes. Coexistence between SA and PA may be an important step in lung bacterial colonization in CF patients, and preventing this phenomenon could participate in the adapted or even personalized management of CF patients.

## Data Availability Statement

All datasets generated for this study are included in the article/Supplementary Material.

## Ethics Statement

All the strains and clinical information used in this study were collected as part of the periodic monitoring of patients at the Hospices Civils de Lyon. As the study is retrospective and non-interventional neither ethics committee approval nor written informed consent were required under local regulations.

## Author Contributions

PB, AD-J, and KM contributed to the conception and design of the study. PR, CM, and FV contributed to design of the study. PB and LC conducted the experiments. SB conducted and analyzed all the statistics. MB and AD-J collected the data. All the authors contributed to the manuscript and approved the submitted version.

### Conflict of Interest

The authors declare that the research was conducted in the absence of any commercial or financial relationships that could be construed as a potential conflict of interest.
