# Supplementary material for: Impact of Coexistence Phenotype Between Staphylococcus aureus and Pseudomonas aeruginosa Isolates on Clinical Outcomes Among Cystic Fibrosis Patients
Source: Front Cell Infect Microbiol. 2020 Jun 3;10:266. doi: 10.3389/fcimb.2020.00266 (PMC7285626; doi:10.3389/fcimb.2020.00266)
Supplement: Supplementary file 1 [file Data_Sheet_1.docx]

# Supplementary data


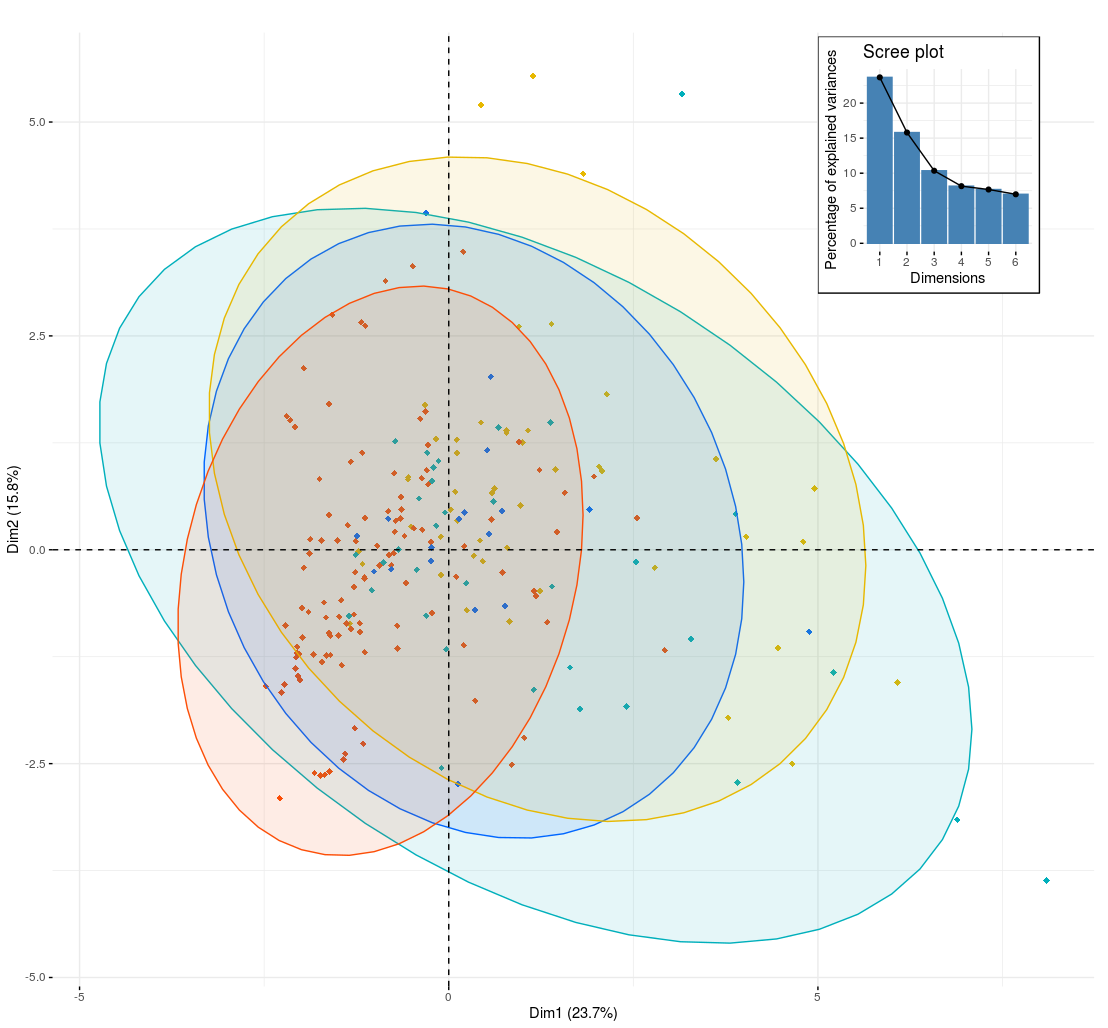


Fig. S1: Individual factor map from FAMD based on 212 patients using the two first principal components. Patients are colored according to their infection type, SA mono-infected (red), PA mono-infected (yellow), SA+PA coinfection in a competition state (blue), SA+PA coinfection in a coexistence state (cyan). Associated eigenvalue plot is shown at the top right.

Table S1: Patients characteristics at the time of the study

| Baseline characteristics | Numbers of patients | Mean ± SD or Percentage |
| --- | --- | --- |
| Age (years) | 212 | 21.70 ± 12.09 |
| ≥18 years | 124/212 | 58.49% |
| Sex, male | 109/212 | 51.42% |
| Genotype |  |  |
| Moderate | 41/212 | 19.34% |
| Severe | 171/212 | 80.66% |
| BMI (kg/m^2^) | 212 | 19.03 ± 2.97 |
| Undernourishment | 16/212 | 7.55% |
| Oral food supplementation | 54/212 | 25.47% |
| Enteral nutrition | 8/212 | 3.77% |
| Pancreatic insufficiency | 196/212 | 92.45% |
| CF-related diabetes | 36/212 | 16.98% |
| Cirrhosis | 9/212 | 4.25% |
| Hospitalizations during study period | 55/212 | 25.94% |
| Number |  | 1.96 ± 1.17 |
| Length (days) |  | 16.38 ± 18.28 |
| Exacerbations* | 84 | 0.78 ± 1.24 |
| FEV1 (% predicted) | 212 | 73.27 ± 25.12 |

BMI: body mass index
FEV1: forced expiratory volume in one second
* Corresponding to total number of exacerbations

Table S2: P-values for categorical variables comparisons between *S. aureus* mono-infected (SA), *P. aeruginosa* mono-infected (PA) and co-infected groups (SA+PA). Fisher’s exact test corrected with a Bonferroni method was used.

|  | PA vs SA | SA+ PA vs SA | SA+PA vs PA |
| --- | --- | --- | --- |
| Sex | - | - | - |
| Genotype | - | - | - |
| Undernourishment | - | - | - |
| Oral food supplementation | - | - | - |
| Enteral nutrition | - | - | - |
| Pancreatic insufficiency | - | - | - |
| CF-related diabetes | 0.0031 | 0.0169 | ns |
| Liver cirrhosis | - | - | - |

Table S3: P-values for categorical variables comparisons between *S. aureus* mono-infected (SA), *P. aeruginosa* mono-infected (PA), co-infected in a coexistence (Coex) state and co-infected in a competition (Comp) state group.

|  | Coex vs SA | Comp vs SA | Coex vs PA | Comp vsPA | Comp. vs Coex |
| --- | --- | --- | --- | --- | --- |
| Sex | - | - | - | - | - |
| Genotype | - | - | - | - | - |
| Undernourishment | - | - | - | - | - |
| Oral food supplementation | ns | ns | ns | ns | ns |
| Enteral nutrition | - | - | - | - | - |
| Pancreatic insufficiency | - | - | - | - | - |
| CF-related diabetes | 0.0164 | ns | ns | ns | ns |
| Liver cirrhosis | - | - | - | - | - |
